# Supplementary material for: Case management programs for people with complex needs: Towards better engagement of community pharmacies and community-based organisations
Source: PLoS One. 2021 Dec 8;16(12):e0260928. doi: 10.1371/journal.pone.0260928 (PMC8654230; doi:10.1371/journal.pone.0260928)
Supplement: S1 File — (DOC) [file pone.0260928.s001.doc]

**INTERVIEW GUIDE**

Interview or focus group reference number: ____________ Date: _________________

Place: __________________________________________ Time: ________________

Interviewer: _____________________________________

**Preamble**

As a [type of interviewee], you are likely to be solicited by case managers. We wish to meet with you to better understand your opinion on case management programs and to learn more about…

- *Program directors and managers*:

…better understand your opinion on the case management program in your IUHSSC (Integrated University Health and Social Services Centres) or in your HSSC (Health and Social Services Centre);

- *Service coordinators and case managers:*

…better understand your opinion on case management programs and learn more about the characteristics you believe have a positive impact on the use of health services by the patients who receive them and on their experience of care. We also wish to understand your needs relating to administrative or clinical support;

- *Physicians:*

…better understand your opinion on case management programs and learn more about the most relevant mechanisms for establishing and maintaining communication between the case manager and the medical clinic;

- *Patients*:

…better understand your opinion on case management programs and learn more about the services you receive under this program, the strengths and areas for improvement as well as the elements of the program that you think have a positive impact on your quality of life, your care experience and your use of health services;

- *Community partners:*

…better understand your opinion on case management programs and learn more about the most relevant mechanisms for establishing and maintaining communication between the case manager, pharmacists/community organizations and other healthcare professionals such as yourself.

Do you have any questions or need clarification regarding high users, case management practice, or the individualized services plan?

**Questions intended specifically for clinicians and community partners**

Can you tell us about the services that you offer?

Can you tell us about the characteristics of the people who use your services?

Please share your experience of dealing with vulnerable people suffering from several chronic diseases as well as mental health and/or psychosocial problems.

Please tell us about the challenges you face in your practice with these people.

Can you tell us about the organization of services for vulnerable people suffering from several chronic diseases as well as mental health and/or psychosocial problems?

How would you describe your access to the health network to meet the specific needs of the people we have just spoken of?

What challenges do you encounter in your practice when it comes time to interact with other healthcare professionals and with other organizations?

What do you think is the best way to integrate the different partners into the frontline network?

What are your views on the integration of case management into the different departments of the IUHSSC and in primary care clinics?

Have you ever been contacted by your territory’s case manager? If so, can you tell us about your experience?

In a situation where confidentiality is an issue, what would be the best way to communicate with the case manager? And with other professionals?

Would you like to participate in individualized services plans if you were asked to do so? Why?

Please tell me about the best interactions that the case manager could have with you. How would you like the case manager to consult you?

Are you able to initiate intervention plans or individualized services plans? If not, who can you consult?

Do you have any success stories to share with us in connection with your participation in the case management program or an individualized services plan?

What is the best strategy that the case manager could use to talk to you about the high users under your care?

When it comes to following up on files, how would you like to communicate with the case manager?

What challenges have you encountered so far in implementing case management?

What is your take on the cases where referrals have led to “revolving doors”? How do patients transition between the different partners within your local service network?

What kind of clinical support would you need?

When thinking of the clientele and case management programs, which characteristics have a positive impact on the use of services and the patient care experience?

What kind of administrative support would you need?

**Questions intended specifically for directors and managers**

What is your management’s role in the deployment of case management services?

What is your role as part of this deployment?

How do you perceive the collaboration across the various departments of the IUHSSC with regard to managing high users in a context where several departments are involved? What could be done to promote inter-program communication?

How can your department provide services to clients whose needs are less clearly defined in the criteria?

What type of structure could the IUHSSC develop in keeping with its vision of the continuum of care to improve coordination and services?

What are the next steps in the development of the IUHSSC’s structure within the continuum of care to improve coordination and services and what is its orientation?

Are you prepared to release members of your department to participate in case management?

In your opinion, how do family physicians view case management programs? In what areas do you feel there is resistance if applicable? Who are the allies?

**Questions intended specifically for patients**

In the context of the services you receive, how are your needs taken into account? Can you give me an example?

In the context of the services you receive, how are the needs of your loved ones taken into account? Can you give me an example?

In the context of the services you receive, what is communication like between you and your case manager? What do you discuss when you communicate with each other? What are the response times when you ask questions? Can you give me an example?

In the context of the services you receive, what is communication like between your loved ones and your case manager? Can you give me an example?

What is your connection between your case manager and the healthcare providers with whom you interact (family physician, nurse, pharmacist, social worker, others)?

Do you have access to information about your health and the services you receive? If so, how do you access this information? If not, why do you think that is?

When you give permission for your loved ones to be informed, how can they access the information about your health and the services you receive?

In what way are you involved in decisions about your health and the services you receive as part of the case management program (taking into account your preferences and wishes regarding these services, care planning, decision-making)? And how are your loved ones involved?

Have you ever participated in an individualized services plan in the presence of the healthcare providers involved in your healthcare and services? If so:

- How did this meeting unfold?
- How were you involved and at what stage?
- Did the following people attend the individualized services plan meeting:
- Your loved ones?
- Your family physician?
- Your nurse?
- Your pharmacist?
- Your social worker?
- If so, did they follow up on the individualized services plan with you?
- If not, why do you think they were absent?

When you meet a new healthcare provider or deal with a new department, how does the meeting unfold?

Are you informed that you are going to meet a new person, deal with a new department, and are you informed of the purpose of the meeting? Please explain.

- Are the new healthcare providers aware of your file?
- Do you have to repeat your medical history? Please explain.
